# European Code Against Cancer, 5th edition – occupational exposures and cancer

**DOI:** 10.1002/1878-0261.70174

**Published:** 2026-01-16

**Authors:** Sylvia H. J. Jochems, Nadia Vilahur, Martie van Tongeren, Maria Albin, Isabelle Baldi, Dario Consonni, Quentin Crowley, Béatrice Fervers, Rüdiger Greinert, Gerard Hoek, Martin Röösli, Hajo Zeeb, Joachim Schüz, Ariadna Feliu, Erica D'Souza, David Ritchie, Carolina Espina, Hans Kromhout

**Affiliations:** ^1^ Institute for Risk Assessment Sciences Utrecht University the Netherlands; ^2^ European Agency for Safety and Health at Work (EU‐OSHA) Bilbao Spain; ^3^ Centre for Occupational and Environmental Health, School of Health Sciences University of Manchester UK; ^4^ Institute for Environmental Medicine Karolinska Institutet Stockholm Sweden; ^5^ Department of Laboratory Medicine Lund University Sweden; ^6^ Inserm U1219, Bordeaux Population Health Research Centre, EPICENE team University Bordeaux France; ^7^ Service Santé Travail Environnement, Pôle de Santé Publique CHU de Bordeaux France; ^8^ Occupational Health Unit Fondazione IRCCS Ca' Granda Ospedale Maggiore Policlinico Milan Italy; ^9^ Geology, School of Natural Sciences Trinity College Dublin Ireland; ^10^ Trinity Centre for the Environment Trinity College Dublin Ireland; ^11^ Department of Prevention Cancer Environment Centre Léon Bérard Lyon France; ^12^ Inserm U1296, “Radiation: Defense, Health and Environment” Lyon France; ^13^ Department of Molecular Cell Biology, Skin Cancer Center Elbe Kliniken Buxtehude Germany; ^14^ Swiss Tropical and Public Health Institute (Swiss TPH) Allschwil 4123 Switzerland; ^15^ University of Basel Basel 4001 Switzerland; ^16^ Department of Prevention and Evaluation Leibniz–Institute for Prevention Research and Epidemiology–BIPS GmbH Bremen Germany; ^17^ Environmental and Lifestyle Epidemiology Branch International Agency for Research on Cancer Lyon France; ^18^ Department of Primary Care and Public Health, School of Public Health Imperial College London London UK

**Keywords:** Europe, European Code Against Cancer, exposure levels, exposure prevalence, occupational cancer, primary prevention, regulations, workplace

## Abstract

Occupational exposure to cancer‐causing agents is a major, yet preventable, contributor to cancer in Europe and globally. In the European Union (EU), cancer is responsible for nearly half of all work‐related deaths, underscoring the critical need for prevention measures. Effective strategies typically involve regulatory and workplace measures aimed at reducing or eliminating exposure risks. Raising awareness of hazardous workplace exposures is essential to empower individuals, foster a culture of prevention, and support effective regulation. The 5th edition of the European Code Against Cancer (ECAC5) includes a recommendation on how individuals can minimize their cancer risk and highlights the shared responsibilities of workers and employers for occupational safety and health: ‘Inform yourself about cancer‐causing factors at work and call on your employer to protect you against them. Always follow health and safety instructions at your workplace’. Key to ECAC5 is the addition of policy pointers at the governance level to support employers in taking preventive action and improving worker awareness. Strengthening regulatory frameworks and increasing awareness are crucial steps toward reducing the burden of occupational cancer.

## Graphical abstract

The 5th edition of the European Code Against Cancer (ECAC5) updates cancer prevention recommendations on occupational exposures, listed at position 9. Individuals are advised to inform themselves about cancer‐causing factors at work and call on their employer to protect them against these factors. They are also advised to always follow health and safety instructions at their workplace. Policymakers are encouraged to strengthen and enforce occupational health and safety legislation and chemical regulations.

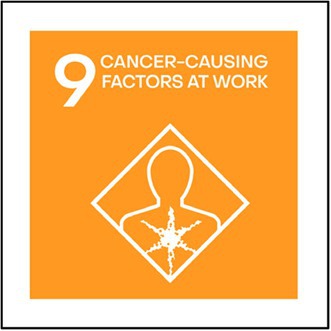

**Abbreviations**
CMRD: carcinogens, mutagens, and reprotoxic substances directive
COPD: chronic obstructive pulmonary disease
DALYs: disability‐adjusted life years
ECAC: European Code Against Cancer
ECAC4: European Code Against Cancer, 4th edition
ECAC5: European Code Against Cancer, 5th edition
EU: European Union
EU‐OSHA: European Agency for Safety and Health at Work
GPP: green public procurement
IARC: International Agency for Research on Cancer
ILO: International Labour Organization
JEM: job‐exposure matrix
KPI: Key Performance Indicator
NCD: non‐communicable disease
NEPSI: European Network on Silica
NGOs: Non‐Governmental Organisations
OEL: occupational exposure limit
OSH: occupational safety and health
PAF: population attributable fraction
PAH: polycyclic aromatic hydrocarbons
PFAS: per‐ and polyfluoroalkyl substances
PFOA: perfluorooctanoic acid
PPE: personal protective equipment
RCS: respirable crystalline silica
SMEs: Small and Medium‐Sized Enterprises
UVR: ultraviolet radiation
WES: European Workers' Exposure Survey
WHO: World Health Organization

## 1 Introduction

The European Code Against Cancer (ECAC) is an initiative of the European Commission aimed at providing clear, evidence‐based recommendations for cancer prevention that are accessible to the general public [1]. The 5th edition (ECAC5) has been coordinated by the International Agency for Research on Cancer (IARC) as part of the World Code Against Cancer Framework to support the development of region‐specific codes tailored to distinct epidemiological and socioeconomic contexts [2]. A scientific methodology has been developed for use in creating any region‐specific codes, including ECAC5, as described in Espina et al. [3].

**Fig. 1:**
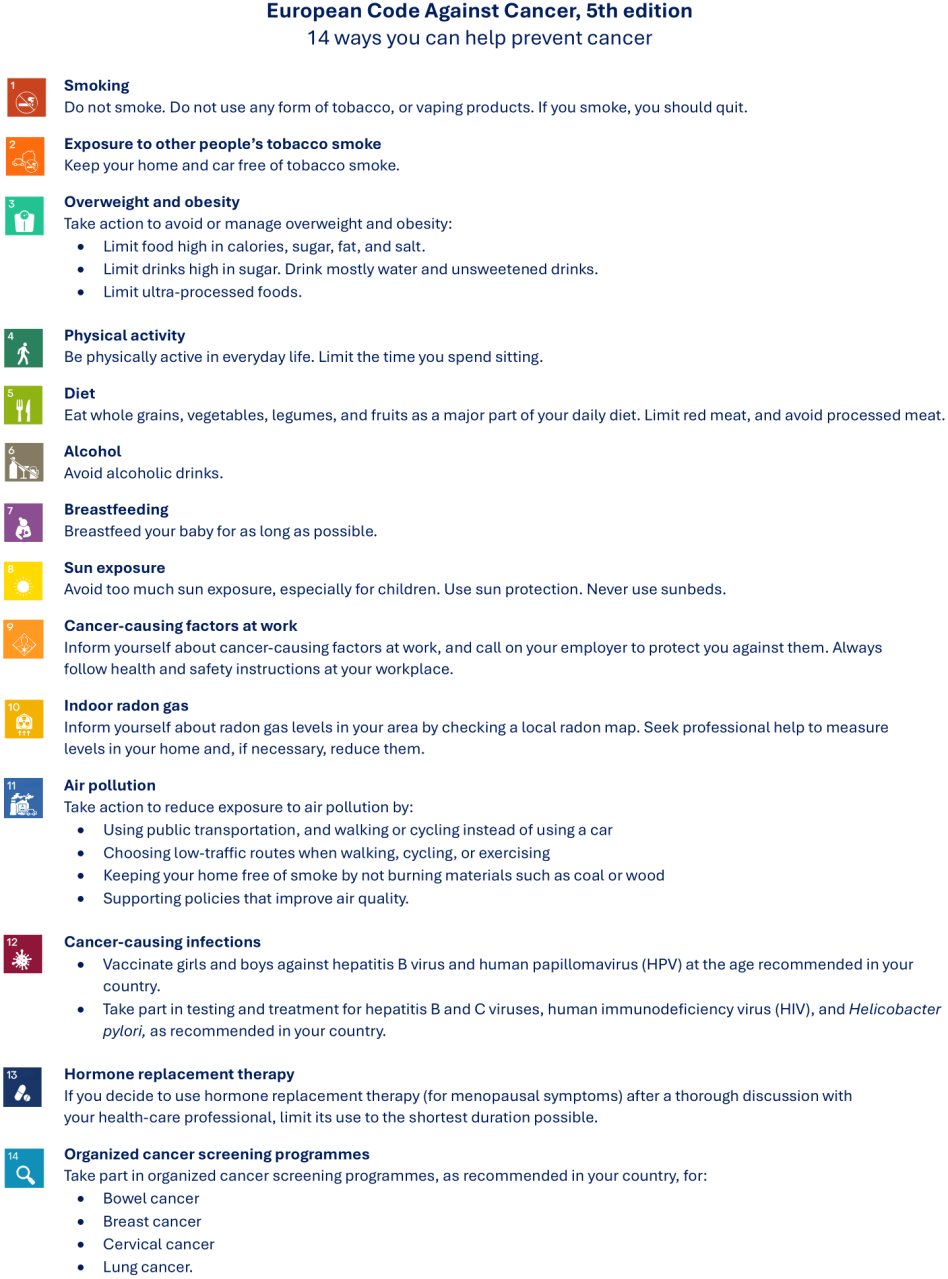
European Code Against Cancer, 5th edition: recommendations for individuals. The 14 recommendations of the European Code Against Cancer, 5th edition (ECAC5) adopted by the Scientific Committee of the ECAC5 project. © 2026 International Agency for Research on Cancer / WHO. Used with permission.

ECAC5 builds on the 4th edition (ECAC4) [1], also coordinated by IARC, by incorporating the latest scientific evidence in cancer prevention into 14 recommendations for individuals, including workers and employers (Fig. 1). For the first time, ECAC5 also targets policymakers, featuring 14 complementary population‐level recommendations that enhance society through governmental support and actions to facilitate adherence to the individual‐level recommendations [4]. This is particularly relevant in the world of work, where major preventative initiatives result from concerted actions involving workers, employers/industries, and governments (Annex S1).

### 1.1 Occupational exposures in the EU

The workplace has historically played a crucial role in identifying carcinogens, as it often involves large groups of individuals exposed to high concentrations of hazardous substances over extended periods, sometimes spanning their entire working lives [5]. Workplace exposure is a key priority for cancer prevention, as many workers encounter carcinogens at levels far higher than those in the general environment. An estimated 80% of workers are exposed to more than one carcinogen at some point of their working lives [6].

### 1.2 EU legislative framework regulating occupational exposures

One of the first European Union (EU)‐wide attempts to address occupational exposure to hazardous substances—though not specifically focused on carcinogens—was Directive 80/1107/EEC [7], which laid the groundwork for later, more comprehensive legislation such as the Occupational Safety and Health (OSH) Framework Directive 89/391/EEC [8] and the EU directive dedicated to occupational exposure to carcinogens, Directive 90/394/EEC [9]. The latter was later codified and expanded as Directive 2004/37/EC [10] which subsequently also incorporated reprotoxic substances, and which is regularly amended and remains the main EU legal framework regulating occupational exposure to carcinogens. In parallel, certain substances, most notably asbestos, are regulated in the EU under substance‐specific legislation, such as Directive 2009/148/EC on the protection of workers from the risks related to exposure to asbestos at work [11].

### 1.3 Monitoring initiatives and exposure prevalence

To address these risks, important initiatives have been set up in the EU such as the ‘Roadmap on Carcinogens’ [12]. The initiative aims to increase awareness of the risks associated with exposure to carcinogen at the workplace, support the implementation of measures to reduce or eliminate hazardous exposures, and foster collaboration between national and EU‐level partners to enhance prevention [12].

Monitoring helps identifying priorities for interventions and to establish effectiveness of interventions. Finland was among the first countries to take action, establishing the Finnish Register of Workers Exposed to Carcinogens (ASA Register) in 1979 [13], which required employers to report workers exposed to carcinogens. In line with this evolving legislation, national systems have been implemented to monitor occupational exposures in various other countries such as Italy [14] and France [15]. The French SUMER register, while not a longitudinal register, partly supports the directive's objectives by providing recurring, descriptive, cross‐sectional data on occupational exposures through national surveys [16]. Additionally, several European exposure studies provided estimates of prevalences of occupational exposure to carcinogens during the 1990s and early 2000s based on available data and exposure measurements with expert assessments and workplace measurements provided valuable resources to improve occupational exposure estimation, risk assessment, and workplace safety interventions [17, 18].

**Fig. 2:**
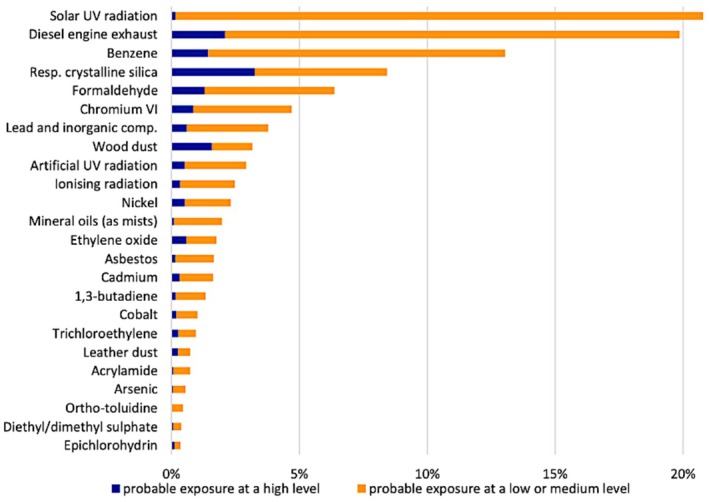
Percentage of workers probably exposed to the 24 cancer risk factors included in the European Workers' Exposure Survey (WES), by level of exposure (% of all workers). The blue bars present the percentage of workers probably exposed to cancer risk factors at a low or medium level. The orange bars present the percentage of workers probably exposed to cancer risk factors at a high level. Figure obtained from EU‐OSHA (2023), Occupational cancer risk factors in Europe—first findings of the Workers' Exposure Survey [19].

In the early 1990s, an estimated 20 to 30 million workers (~a quarter of the total workforce) were exposed to carcinogens [17]. More recently data became available from the European Agency for Safety and Health at Work (EU‐OSHA), from the 2022 to 2023 European Workers' Exposure Survey (WES). This interviewer‐administrated survey was carried out in six EU countries (Finland, France, Germany, Hungary, Ireland, and Spain) covering workers aged ≥15 years across all sectors of occupation. The results showed that prevalence of exposure to carcinogens was much higher than previous estimates: about 47% of the workforce were judged to be exposed to at least one of the 24 cancer risk factors considered in the survey [19]. The sectors with the highest exposures were construction, manufacturing, and agriculture, with the most frequent exposures including solar and artificial UV radiation, ionizing radiation, diesel engine exhaust emissions, benzene, respirable crystalline silica (RCS), formaldehyde, hexavalent chromium, lead and its inorganic compounds, and wood dust [19], as shown in Fig. 2. These higher prevalence estimates are likely to be due to differences in methodology, including exposure definitions, time frame, and regulatory coverage.

Lower‐paid jobs and/or physically demanding jobs often carry greater risks, disproportionately affecting the youngest and oldest individuals, men and women, and migrant workers [20]. These groups are more likely to occupy precarious or high‐risk roles with limited access to safety training. Socioeconomic status further impacts health outcomes including cancer, as hazardous workplace exposures are more common in lower‐paying jobs, contributing to disparities in occupational disease risk and life expectancy [21].

### 1.4 Cancer burden in the EU attributable to occupational exposures

Occupational exposure to carcinogens remains a major public health concern, contributing significantly to overall cancer incidence and mortality in the region. Cancer is the leading cause of work‐related mortality in the EU, with an estimated 45.6% of annual occupational deaths attributed to work‐related cancers [22]. In 2019, approximately 176 000 work‐related deaths occurred across Europe, with around 80 250 attributed to occupational cancers [20]. Reducing occupational cancer is a key priority in the EU, where efforts increasingly focus on prevention as well as improved survival. However, individuals diagnosed with occupational cancers often face prolonged treatment and recovery periods, which can limit their ability to work and may lead to job loss and increase the risk of disengagement from work. This results in economic consequences at societal level, including productivity losses and increased healthcare expenditures. A study by the European Trade Union Institute [23] estimated that the economic burden of cancer resulting from past occupational exposure to selected carcinogenic substances in the EU is considerable. In risk assessment, cancer incidence is often the preferred measure, as it provides a direct estimate of disease occurrence linked to occupational exposures, independent from advances in treatment and survival in high‐income countries. Addressing occupational cancer risk requires a comprehensive approach that accounts not only for the immediate health impacts but also for the broader socioeconomic consequences of the disease.

It has been estimated that occupational cancers account for approximately 2–8% of all cancer cases worldwide [24]. The EU‐OSHA estimated that in 2023 there were over 37 000 new cases of cancer due to occupational exposures [20]. The most frequent were lung cancer (15 272 cases) and mesothelioma (14 914 cases) [20]. Bladder cancer was the third most common, with approximately 2500 cases [20]. Notably, for certain cancer types with strong occupational risk factors, the proportion attributable to occupational exposure can be higher. For example, a French study assessing the general population in 2015 estimated that about 71% of mesothelioma, 15% of lung cancer, 17% of nasopharyngeal carcinoma, and 25% of bladder cancer cases were attributable to occupational exposure [25]. However, the total burden remains difficult to determine. Contributing factors include the long latency of many occupational cancers, challenges in surveillance, uncertainties regarding the carcinogenicity of workplace exposures, and uncertainty about the number exposed (i.e., the prevalence of exposure to carcinogens in the workplace) [26]. Regarding occupational cancer deaths, Western Europe reported the highest number (92 443 deaths; 20.77 deaths per 100 000 in 2023) [27]. Globally, the Global Burden of Disease Study (GBD 2016) estimated that the overall population attributable fraction (PAF) for occupational carcinogens was 3.9% for deaths and 3.4% for DALYs, with higher numbers in men (5.3%) than in women (2.0%) [28].

These findings highlight the enormous impact of cancer in the EU and globally and reinforce the urgent need for effective preventive strategies to reduce the impact of occupational carcinogens. There is convincing evidence that preventive measures can effectively reduce occupational cancer risks. In the United Kingdom, a substantial decline in mesothelioma incidence has followed reductions in occupational asbestos exposure after banning its use [29]. Similarly, the implementation of smoke‐free workplace laws across Europe has significantly reduced second‐hand smoke exposure, a known cause of lung cancer [30]. These examples demonstrate that targeted policy action can meaningfully reduce occupational cancer risks.

## 2 Recommendations for individuals

### 2.1 Scientific justification for updating the recommendation

The number of known occupational carcinogens has increased over time, highlighting the urgency of substituting hazardous substances where possible and strengthening workplace safety regulations [31]. However, in some cases, the carcinogenicity of suspected substances remains unresolved due to limited human data, and the true burden of occupational cancer is likely underestimated. As more evidence becomes available, authoritative bodies may revise substance classifications; therefore, ECAC recommendations should be periodically updated to reflect such changes [32].

### 2.2 Evidence on the association between occupational exposures and cancer

Agent classifications are critical for identifying occupational carcinogens and guiding prevention strategies. Table 1 shows agents that have been newly classified, reclassified, or reaffirmed as carcinogenic to humans (Group 1 carcinogens) over the past decade, based on accumulating epidemiological, animal, and mechanistic evidence [33]. However, classifications are not static—they evolve as new data emerge [32].

**Table 1:** Overview of occupational exposures and occupations classified as carcinogenic to humans (Group 1 carcinogen) by IARC since ECAC4.

Carcinogenic agent	Cancer types with sufficient evidence in humans	Cancer types with limited evidence in humans	Classification status^a^
**Chemicals and mixtures**			
Acrylonitrile	Lung cancer	Bladder cancer	Reaffirmed
Benzene	Leukemia	Lymphoma Multiple myeloma Lung cancer	Reaffirmed
Pentachlorophenol	Lymphoma Multiple myeloma		Reclassified
1,2‐Dichloropropane	Bile duct cancer		Reclassified
**Industrial processes**			
Acheson process	Lung cancer		Newly classified
**Per‐ and Polyfluoroalkyl substances (PFAS)**			
Perfluorooctanoic acid (PFOA)		Kidney cancer Testicular cancer	Reclassified
**Welding**			
Fumes	Lung cancer	Kidney cancer	Reclassified
Ultraviolet radiation (UVR)	Ocular melanoma		Reaffirmed
**Occupations**			
Firefighter	Mesothelioma Bladder cancer	Lymphoma Colon cancer Prostate cancer Melanoma of the skin Testicular cancer	Reclassified

a Classification status reflects changes since the publication of ECAC4 on occupational exposures and cancer in 2014. This includes agents newly classified as Group 1 carcinogens, those reclassified from a lower group (e.g., 2A or 2B), and previously classified Group 1 carcinogens whose classification was reaffirmed. The table covers Group 1 carcinogens since ECAC4 but is restricted to agents with current occupational use in the EU; historically used agents (e.g., lindane) are not shown.

The Acheson process, used to produce silicon carbide, was classified for the first time as a Group 1 carcinogen in 2014 [34]. The process generates silicon carbide fibers as an unwanted by‐product. Epidemiological studies from Norway and Canada have shown an increased risk of lung cancer among furnace workers exposed to these fibers [35, 36]. While silicon carbide had been evaluated previously, this was the first time the occupational exposure context specific to the Acheson process was classified as carcinogenic to humans.

New evidence has reaffirmed the Group 1 classification of several carcinogens. For example, benzene remains strongly associated with acute myeloid leukemia and shows positive associations with other hematological malignancies [37]. Occupational exposure to benzene occurs mainly in petrochemical industries, rubber manufacturing, and laboratories.

Acrylonitrile, a high‐production‐volume chemical, is primarily used as a monomer in the manufacture of synthetic fibers, plastics, and rubber. Workers are mainly exposed in production facilities. It was reaffirmed as a Group 1 carcinogen based on its association with lung cancer [38].

Ultraviolet radiation (UVR) from welding has also been reaffirmed as a Group 1 carcinogen due to sufficient evidence that it causes ocular melanoma [39]. While ocular melanoma had already been associated with welding in 2012, the specific role of UVR in causing this cancer was confirmed in 2017. Welding fumes were reclassified from possibly carcinogenic to carcinogenic based on sufficient evidence of lung cancer [39].

Several agents previously classified as Group 2A or 2B were reclassified to Group 1 carcinogens based on new evidence since the previous ECAC edition [33]. Pentachlorophenol, previously considered a probable carcinogen, was reclassified due to sufficient evidence linking it to non‐Hodgkin lymphoma [40]. Occupational exposure occurs primarily in wood treatment, pesticide application, and waste incineration.

Lindane, a pesticide banned in the EU since 2007, was reclassified as a Group 1 carcinogen [41] due to its association with non‐Hodgkin lymphoma in pesticide applicators and agricultural workers [42, 43]. Although it is no longer in use in the EU, many cancer cases have long latency periods, making past exposure still relevant now.

1,2‐Dichloropropane, a chlorinated solvent used in industrial printing and chemical manufacturing, was reclassified to Group 1 carcinogen following studies in Japan that showed a strong association with bile duct cancer in printing workers [44]. Although these findings are based on non‐European data, this chemical is still in use in Europe under strict regulatory conditions, highlighting the need for continued exposure monitoring.

Perfluorooctanoic acid (PFOA), part of the per‐ and polyfluoroalkyl substances (PFAS) group, was reclassified from Group 2B to Group 1 carcinogen. Occupational exposure has occurred primarily in chemical manufacturing, especially among fluorochemical‐production workers and firefighters. Inhalation and dermal absorption are the main exposure routes.

Firefighting was classified as an occupational exposure scenario involving Group 1 carcinogens, including asbestos and combustion products [45]. For occupational exposure as a firefighter, there is sufficient evidence for mesothelioma and bladder cancer, and more limited evidence for other cancers including colon, lymphoma, prostate, melanoma, testicular, and thyroid cancer [45]. The findings highlight the importance of improved protective measures and decontamination protocols.

Night shift work is currently classified as probably carcinogenic to humans (Group 2A), based on sufficient evidence in animals and limited evidence in humans, particularly for breast cancer [46]. Given its widespread prevalence and the emerging evidence since the 2019 classification, it should be considered a priority for further research and preventive action.

### 2.3 Presentation of the recommendation

**Fig. 3:**
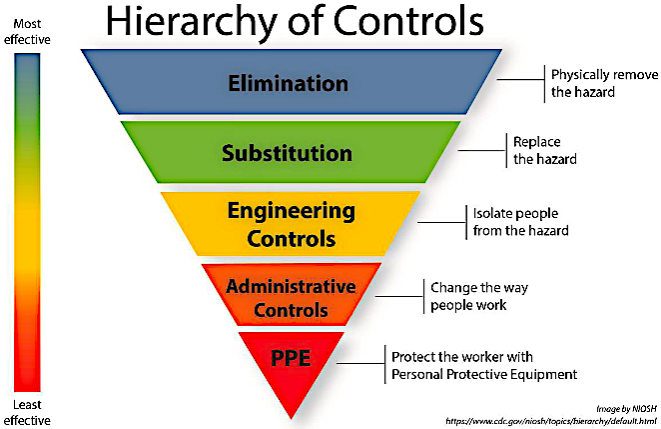
Hierarchy of controls. The hierarchy of controls prioritizes the most effective interventions. The framework consists of five consecutive steps in relation to workplace hazards: elimination, substitution, technical/engineering controls, organizational/administrative controls, personal protective equipment (PPE). Figure obtained from About Hierarchy of Controls, Hierarchy of Controls, CDC, and by NIOSH.

The Hierarchy of Controls is a fundamental framework in OSH, guiding organizations in reducing workers' exposure to hazards and reflected in the text of OSH Directives. Fig. 3 summarizes the Hierarchy of Controls (E‐STOP) [47] and emphasizes the importance of prioritizing risk mitigation strategies.

#### 2.3.1 Hierarchy of controls (E‐STOP)

The hierarchy of controls prioritizes the most effective interventions. The framework consists of five consecutive steps in relation to workplace hazards:

- Elimination
- Substitution
- Technical/Engineering controls
- Organizational/Administrative controls
- Personal Protective Equipment (PPE)

Elimination is the most effective method for protecting workers, as it involves removing the cancer hazard entirely, such as discontinuing the use of a carcinogen or redesigning work processes to eliminate exposure risks. When elimination is not feasible, substitution involves replacing carcinogens or processes with safer alternatives that meet the same or similar requirements of the original hazardous ones. However, it is important to ensure that replacement agents do not pose trade‐offs in terms of other health effects or are insufficiently regulated simply due to a lack of knowledge on their health effects. Engineering controls involve redesigning the workplace to separate workers from hazards. For carcinogens, this can include working in closed systems or using effective local exhaust ventilation systems to remove airborne contaminants, including on‐tool extraction or automated equipment to minimize direct contact with dangerous equipment. These controls can be highly effective because they generally rely less on individual compliance and instead aim to create a safer environment by design. However, their effectiveness still depends on proper implementation and correct use, as misuse can reduce their protective benefits. Administrative controls involve changes in work policies and procedures to reduce risk. These may include rotating job tasks to limit exposure time, enforcing mandatory breaks to reduce fatigue and risk of accidents, or implementing lockout/tagout protocols to ensure equipment is safely deactivated before maintenance. PPE includes respirators, gloves, protective clothing, and eye and face protection, which serve as physical barriers between workers and carcinogenic hazards. However, it is generally considered the least effective control because it relies on proper use, fit, and maintenance and may be burdensome to wear under specific conditions (e.g., hot weather). PPE should always be used in combination with higher‐level controls, such as engineering or administrative measures, to provide comprehensive protection.

Workers can reduce their risk of occupational cancer by following established workplace safety protocols, using protective equipment, and demanding proper hazard control measures from employers (an obligation laid down by OSH legislation). All these elements are brought forward in the new ECAC recommendation. Clear communication about workplace hazards is essential to enable informed decision‐making, but access to information and protective measures is not always equitable. Migrants, younger workers, and those in precarious employment often face higher exposure levels while having fewer resources to mitigate risks.

Promoting equity in occupational cancer prevention requires systemic efforts to ensure that no group is left behind. Low‐wage, temporary, and informal sector workers often lack access to protective measures and are more vulnerable to the consequences of ill health. Similarly, migrants, those with limited language proficiency, or young workers may struggle to understand safety protocols and, as new entrants eager to establish themselves in the labour market, may be more prone to risk‐taking behavior or to accepting unsafe tasks. Tailoring communication, providing training in accessible formats, and involving labor unions and Non‐Governmental Organisations (NGOs) can help close these gaps. Preventing exposure also protects against the economic hardship that may result from long‐term illness or reduced work ability, especially in physically demanding jobs. Facilitating that all workers receive adequate training in a language they can understand, access to protective equipment, and transparent information on the hazards to which they are exposed in their daily work is critical to reducing disparities in occupational cancer risks and health outcomes. Additionally, ensuring employers take responsibility for eliminating or minimizing carcinogenic exposures across all sectors and regions reinforces accountability and promotes fairer outcomes for all workers.

The previous ECAC4 recommendation has been adjusted to emphasize awareness and proactive advocacy for workplace safety, shifting responsibility from just self‐protection to also holding employers accountable [33]. The ECAC5 recommendation on how working individuals can minimize their cancer risk reads:

*Inform yourself about cancer‐causing factors at work and call on your employer to protect you against them. Always follow health and safety instructions at your workplace.*

The recommendation is suitable, actionable, and culturally acceptable across the EU's diverse workforce. It aligns with existing occupational safety and health policies and frameworks, including the EU's ‘Vision Zero’ strategy to eliminate work‐related deaths by 2030 [48], relevant International Labour Organization (ILO) conventions [49], and the 2030 Agenda for sustainable development [50]. Its emphasis on shared responsibility between workers, employers, and public bodies ensures that it is both realistic and implementable without requiring specialist knowledge.

### 2.4 Cobenefits for the prevention of noncommunicable diseases other than cancer with similar risk factors and opportunities for health promotion

Reducing occupational exposure to carcinogens also lowers risks for common work‐related noncommunicable diseases (NCDs) that pose a high health burden in exposed workers. For example, reducing diesel engine exhaust emissions exposure not only decreases lung cancer rates but will also reduce the risk of developing chronic obstructive pulmonary disease (COPD) and exacerbation of asthma [51], and possibly myocardial infarction [52]. Similarly, reducing exposure to solvents and heavy metals will also reduce risk of neurological and reproductive ill health. Integrating a broader approach to primary prevention of NCDs that includes occupational health interventions improves worker well‐being, reduces healthcare burden and costs, and will have economic benefits and a positive impact on society as a whole.

## 3 Recommendations for policymakers

Policymakers in the EU can reduce occupational cancer by strengthening and enforcing occupational health and safety legislation and chemical regulations.

### 3.1 Presentation of the recommendation for policymakers and key stakeholders

Table 2 shows the recommendations for policymakers on occupational exposures developed for the first time in ECAC. All economic sectors, including small and medium‐sized enterprises (SMEs) and the self‐employed, should collaborate with social partners to reduce both the prevalence and the levels of exposure to carcinogens and to track progress with simple indicators. SMEs and the self‐employed should receive targeted support to ensure active participation in these initiatives.

**Table 2:** European Code Against Cancer, 5th edition: recommendations for policymakers on occupational exposures.

Cancer‐causing factors at work	
•	Scale up efforts to enforce existing EU legislation on occupational carcinogens, including compliance with binding occupational exposure limits.
•	Encourage all economic sectors with exposure to carcinogens to work with social partners to develop and implement social dialog agreements for reduction of the prevalence and levels of exposure, and to monitor and publish key performance indicators. Support small and medium‐sized enterprises and self‐employed workers to actively engage with such initiatives.
•	Include specific occupational safety and health (OSH) requirements in the criteria for public procurement, to support the elimination and/or reduction of workers’ exposure to carcinogens in the workplace.
•	Ensure that knowledge on safe work practices and how to prevent exposure to carcinogens is integrated into education programs, including in vocational training.

© 2026 International Agency for Research on Cancer / WHO. Used with permission.

References: • Directive 89/391/EEC on the introduction of measures to encourage improvements in the safety and health of workers at work (OSH “Framework Directive“). *OJEU*. 1989;**L183**:1–8. Available from: https://eur-lex.europa.eu/legal-content/EN/TXT/?uri=CELEX:01989L0391-20081211 [8]. • Directive 2004/37/EC on the protection of workers from the risks related to exposure to carcinogens or mutagens at work. *OJEU*. 2004;**L229**:23–34. Available from: https://eurlex.europa.eu/legal-content/EN/TXT/?uri=CELEX%3A02004L0037-20240408&qid=1721724278253 [10]. • Directive 2009/148/EC on the protection of workers from the risks related to exposure to asbestos at work.*OJEU*. 2009;**L330**:28–36. Available from: https://eur-lex.europa.eu/eli/dir/2009/148 [11]. • Regulation (EU). EU 2023/1542 of 12 July 2023 concerning batteries and waste batteries. *OJEU*. 2023;**L191**:1–102. Available from: https://eur-lex.europa.eu/eli/reg/2023/1542 [60]. • Roadmap on Carcinogens: A European‐wide voluntary action scheme to tackle work‐related cancer. Available from: https://stopcarcinogensatwork.eu [12].

A strong legislative foundation already exists across the EU, notably the Carcinogens, Mutagens, and Reproductive Substances Directive (CMRD; Directive 2004/37/EC) [10], the Directive on asbestos at work (Directive 2009/148/EC) [11], and the Registration, Evaluation, Authorisation and Restriction of Chemicals (REACH) regulation [53]. These policies align with Europe's Beating Cancer Plan [54] and international frameworks, such as the ILO's Fundamental Conventions on Occupational Safety and Health [49], the World Health Organization's (WHO) Global Plan of Action on Workers' Health [55], and the NCD Best Buys [56]. However, enforcement remains inconsistent across countries and sectors, requiring stronger oversight and standardized frameworks to ensure uniform worker protection. The burden of work‐related cancers extends beyond mortality and morbidity, affecting quality of life, productivity, and healthcare costs.

To enhance individual health prevention measures in the workplace, policymakers should:

- Enforce existing EU legislation on occupational carcinogens, especially binding OELs. Implementing Directive 2004/37/EC [10] requires coordinated action at national and EU level. Sector‐specific risk assessment tools, practical guidance on chemical risk management, and accessible training packages can support employers in meeting legal obligations. Strengthen guidance for labor inspectorates to improve oversight and compliance.
- Foster cross‐sector collaboration to reduce exposure. Support voluntary agreements with clear targets and reporting (e.g., Roadmap on Carcinogens [12], European Network on Silica (NEPSI) for silica [57], and Formacare for formaldehyde [58]). Provide tailored support so SMEs and the self‐employed can participate.
- Integrate occupational safety and health requirements into public procurement and ensure it reaches subcontractors. Inspired by legally binding Green Public Procurement (GPP) criteria and EU regulations that integrate sustainability and safety into procurement frameworks [59, 60], occupational safety and health‐related conditions should be incorporated into public purchasing processes. Include flow‐down occupational safety and health clauses to all subcontractors and make the main contractor responsible for their compliance, verified through site‐access checks (training/inductions) and simple exposure‐control records. Compliance should extend to subcontractors and their workers, particularly in sectors where outsourcing is common, such as construction.
- Add occupational safety and health to education and vocational training, especially for new and young workers [61]. Embed basic exposure‐prevention content in curricula and apprenticeships. Use practical tools on safe handling, storage, and control measures. Examples include the EU‐OSHA program ‘Healthy Workplaces for All Ages’ [62] and the Online Interactive Risk Assessment (OiRA) tools developed to help integrate risk prevention in training and small businesses [63]. Employers should define essential occupational safety and health competencies and update them regularly.
- Promote public–private partnerships to speed up safer technologies and nonregrettable substitution of hazardous agents. Encourage collaboration between industry, research institutions, and authorities to test and adopt safer alternatives and processes. For example, the European Partnership for the Assessment of Risks from Chemicals (PARC) brings together public authorities, research institutions, and industry to support innovation in chemical risk assessment and promote the substitution of hazardous substances [64].

Clear, well‐enforced regulations and social dialog protect workers and help them raise concerns about health risks. Regulators should ensure that employers prioritize measures at the top of the hierarchy of controls (elimination, substitution, engineering) to prevent or reduce exposures at the source; the remaining risk is smaller, so PPE becomes unnecessary or a more effective last line of defense.

### 3.2 Feasibility and resources required to implement the recommendation

Implementing occupational health and safety policies requires coordinated efforts, sufficient resources, and strong enforcement to ensure effectiveness. Experience from EU countries shows that stringent regulations, enhanced monitoring, and targeted interventions can reduce occupational exposure to carcinogens and improve worker health outcomes [65].

The European Network on Silica (NEPSI) Agreement, signed in 2006, aims to reduce exposure to RCS [57]. Supported by the European Commission, it includes a Good Practice Guide developed with input from national health institutes, and mandates exposure monitoring, training, and health surveillance. Compliance is tracked through key performance indicators (KPIs), such as regulatory compliance rates, policy adherence, and training and awareness completion rates, with biennial reporting on exposure‐control measures and implementation trends. While NEPSI provides a framework for risk management, several studies offer quantitative exposure data [66, 67], complementing NEPSI's self‐reported indicators. Combining social dialog agreements like NEPSI with accessible measurement data from workplaces helps refine regulations and reduce long‐term health risks.

Although the implementation of such policies requires upfront investment, prevention should not be seen as a mere economic burden. For instance, evidence from an occupational health intervention on reducing heat stress prevention shows that targeted preventive actions can lead to economic benefits by reducing disease burden, sickness absence, and associated productivity losses [68]. Similarly, a study on asbestos‐related diseases indicates that the societal costs of occupational cancers, including treatment, productivity losses, and premature mortality, far exceed the costs of preventive interventions [69]. Even in the absence of specific cost estimates for all occupational cancers, these examples underscore that prevention can lead to a healthier workforce, fewer absences, and ultimately higher economic output.

## 4 Conclusions

Despite being preventable, occupational exposures to known human carcinogens remain widespread in Europe and call for increased attention and sustained action. While legislation and risk management measures have helped reduce exposure levels in EU workplaces, gaps in enforcement and emerging new hazards call for continued vigilance. To reduce the burden of occupational cancers, it is critical to strengthening workplace protection reaching all workers, improve exposure monitoring, and ensure compliance with binding OELs through effective risk management measures. Aligning prevention efforts with robust policies can create safer workplaces, especially for vulnerable groups, by placing occupational safety and health at the center, while also yielding economic and public health benefits. Lasting progress can only be achieved through awareness and collective commitment and decisive action by governments, industries, employers, and workers.

## Conflict of interest

DC served as a consultant for the judiciary in litigations concerning asbestos‐related diseases. The authors SHJJ, NV, MvT, MA, IB, QC, BF, RG, GH, MR, HZ, JS, AF, ED, DR, CE, and HK declare no conflict of interest. Where authors are identified as personnel of the International Agency for Research on Cancer/World Health Organization, the authors alone are responsible for the views expressed in this article and they do not necessarily represent the decisions, policy or views of the International Agency for Research on Cancer/World Health Organization.

## Author contributions

SHJJ and HK were responsible for writing the first version of the manuscript. All authors gave critical revisions on the intellectual content of the manuscript and approved the final manuscript.

## Supporting information


**Annex S1.** European Code Against Cancer, 5th edition. © 2026 International Agency for Research on Cancer / WHO. Used with permission.

## Data Availability

The data that supports the findings of this study are available in Figs 1, 2, 3 and Tables 1 and 2 and the Supporting Information of this article.
